# Morphology-based radiological-histological correlation on ultra-high-resolution energy-integrating detector CT using cadaveric human lungs: nodule and airway analysis

**DOI:** 10.1007/s00330-025-11756-1

**Published:** 2025-06-26

**Authors:** Akinori Hata, Masahiro Yanagawa, Keisuke Ninomiya, Noriko Kikuchi, Masako Kurashige, Daiki Nishigaki, Shuhei Doi, Kazuki Yamagata, Yuriko Yoshida, Ryo Ogawa, Yukiko Tokuda, Eiichi Morii, Noriyuki Tomiyama

**Affiliations:** 1 Department of Diagnostic and Interventional Radiology, Graduate School of Medicine, Osaka University, 2-2 Yamadaoka, Suita, Osaka 5650871 Japan; 2 Department of Radiology, Minoh City Hospital, 7-1 Kayano 5 chome, Minoh City, Osaka 562-0014, Osaka, 5650871 Japan; 3 Department of Pathology, Graduate School of Medicine, Osaka University, 2-2 Yamadaoka, Suita, Osaka 5650871 Japan

**Keywords:** Lung, Multiple pulmonary nodules, Bronchioles, X-ray computed tomography

## Abstract

**Objectives:**

To evaluate the depiction capability of fine lung nodules and airways using high-resolution settings on ultra-high-resolution energy-integrating detector CT (UHR-CT), incorporating large matrix sizes, thin-slice thickness, and iterative reconstruction (IR)/deep-learning reconstruction (DLR), using cadaveric human lungs and corresponding histological images.

**Materials and methods:**

Images of 20 lungs were acquired using conventional CT (CCT), UHR-CT, and photon-counting detector CT (PCD-CT). CCT images were reconstructed with a 512 matrix and IR (CCT-512-IR). UHR-CT images were reconstructed with four settings by varying the matrix size and the reconstruction method: UHR-512-IR, UHR-1024-IR, UHR-2048-IR, and UHR-1024-DLR. Two imaging settings of PCD-CT were used: PCD-512-IR and PCD-1024-IR. CT images were visually evaluated and compared with histology.

**Results:**

Overall, 6769 nodules (median: 1321 µm) and 92 airways (median: 851 µm) were evaluated. For nodules, UHR-2048-IR outperformed CCT-512-IR, UHR-512-IR, and UHR-1024-IR (*p* < 0.001). UHR-1024-DLR showed no significant difference from UHR-2048-IR in the overall nodule score after Bonferroni correction (uncorrected *p* = 0.043); however, for nodules > 1000 μm, UHR-2048-IR demonstrated significantly better scores than UHR-1024-DLR (*p* = 0.003). For airways, UHR-1024-IR and UHR-512-IR showed significant differences (*p* < 0.001), with no notable differences among UHR-1024-IR, UHR-2048-IR, and UHR-1024-DLR. UHR-2048-IR detected nodules and airways with median diameters of 604 µm and 699 µm, respectively. No significant difference was observed between UHR-512-IR and PCD-512-IR (*p* > 0.1). PCD-1024-IR outperformed UHR-CTs for nodules > 1000 μm (*p* ≤ 0.001), while UHR-1024-DLR outperformed PCD-1024-IR for airways > 1000 μm (*p* = 0.005).

**Conclusions:**

UHR-2048-IR demonstrated the highest scores among the evaluated EID-CT images. UHR-CT showed potential for detecting submillimeter nodules and airways. With the 512 matrix, UHR-CT demonstrated performance comparable to PCD-CT.

**Key Points:**

***Question*** *There are scarce data evaluating the depiction capabilities of ultra-high-resolution energy-integrating detector CT (UHR-CT) for fine structures, nor any comparisons with photon-counting detector CT (PCD-CT).*

***Findings*** *UHR-CT depicted nodules and airways with median diameters of 604* µm and 699 µm, showing no significant difference from PCD-CT with the 512 matrix.

***Clinical relevance*** *High-resolution imaging is crucial for lung diagnosis. UHR-CT has the potential to contribute to pulmonary nodule diagnosis and airway disease evaluation by detecting fine opacities and airways.*

**Graphical Abstract:**

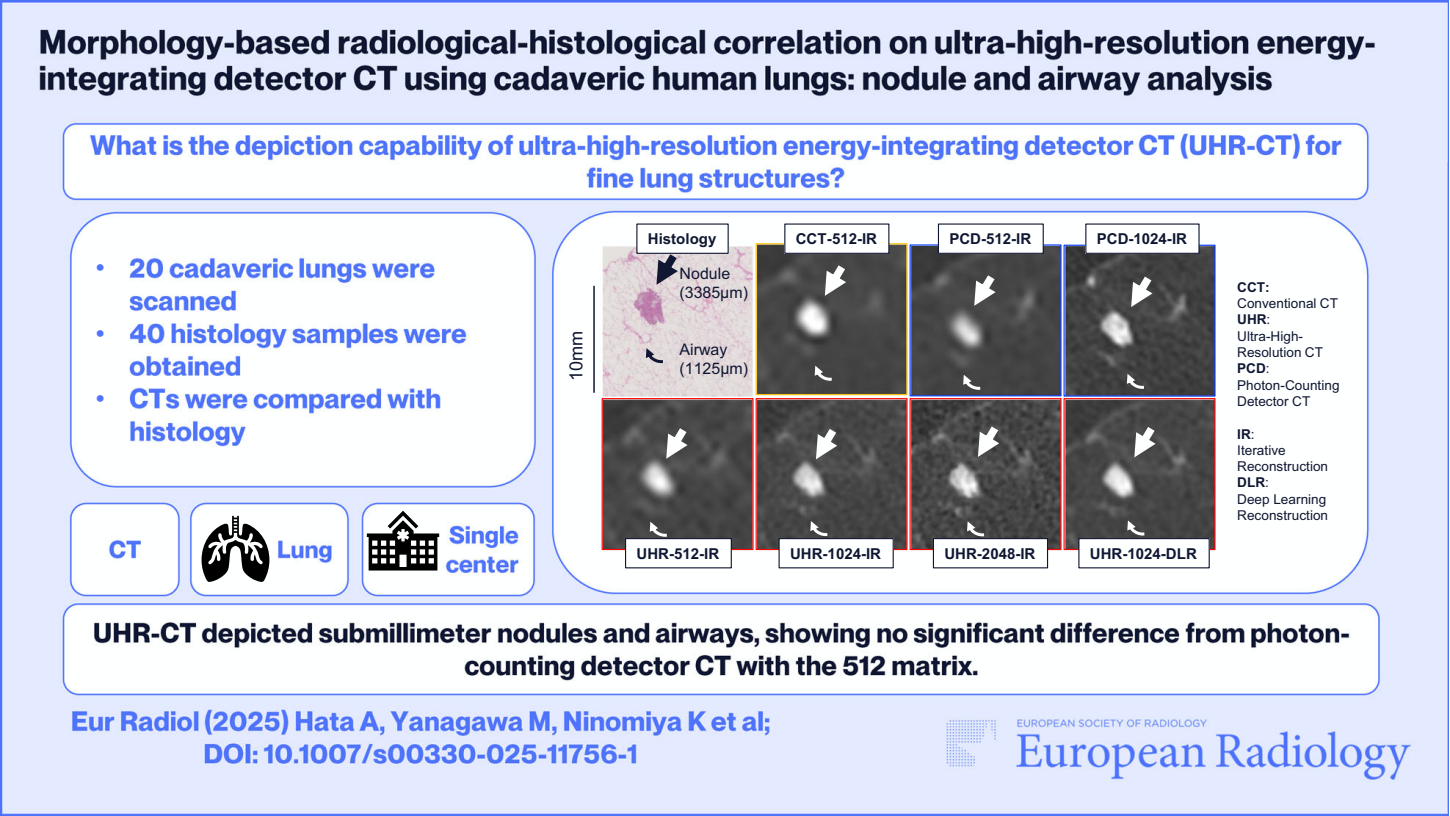

## Introduction

CT imaging has achieved a higher spatial resolution due to advancements in technology. An ultra-high-resolution energy-integrating detector CT (UHR-EID-CT), which features a smaller detector element size and x-ray tube focus compared to the conventional CT (CCT), became clinically available in 2017 [1, 2]. While CCT offers a spatial resolution of 0.23–0.35 mm [3, 4], UHR-CT achieves a resolution of 0.14 mm and better image quality in the lungs [1, 2].

We previously conducted a study comparing UHR-CT with CCT using cadaveric human lungs and reported that UHR-CT provided superior depiction of findings such as nodules, ground-glass opacities, consolidation, emphysema, interlobular septal thickening, bronchovascular bundle thickening, bronchiectasis, and honeycombing [1]. Furthermore, using similar cadaveric human lungs, we compared images reconstructed with different matrix sizes available in UHR-CT (512, 1024, and 2048) and demonstrated that images with a 2048 matrix exhibited superior quality [2]. However, these studies subjectively evaluated UHR-CT and CCT without a more reliable reference standard, such as histological images, and did not assess the precise size of the lesions. Previous reports have also highlighted the significant role of the bronchus cutoff sign in UHR-CT as a predictor of invasion in lung adenocarcinoma [5], improved visualization of the Adamkiewicz artery [6], and enhanced depiction of middle ear structures [7].

Iterative reconstruction (IR) is widely used in clinical practice to enhance CT image quality [8, 9]. Deep-learning-based reconstruction (DLR) methods utilizing deep convolutional neural networks have been developed to achieve further improvements in image quality [8, 9]. In general, the use of deep-learning technology often results in algorithms becoming “black boxes,” raising concerns about whether DLR truly reflects the actual anatomical structures. Although DLR improves image quality and lesion detectability [10], no study has compared DLR with histological images to assess whether DLR accurately represents the microscopic structure of the lungs.

Photon-counting detector CT (PCD-CT), which utilizes a different approach compared to the EID-CT, became clinically available in 2021 [11–13]. PCD-CT achieves a spatial resolution of 0.11 mm [14]. We previously demonstrated that PCD-CT provides superior visualization compared to conventional EID-CT by imaging cadaveric human lungs and comparing them with histological images as reference standards [15].

The evaluation of pulmonary nodules on CT plays a crucial role in the early detection and management of lung diseases, particularly lung cancer. Accurate characterization of nodules based on size, shape, density, and growth patterns enables risk stratification and informs clinical decision-making [16]. Given the potential impact on patient outcomes, precise CT assessment of pulmonary nodules remains an essential component of thoracic radiology and oncologic evaluation.

The purpose of this study was to evaluate the depiction capability of fine lung nodules and airways using high-resolution imaging settings on UHR-EID-CT, incorporating large matrix sizes, thin-slice thickness, and IR/DLR, in comparison with conventional EID-CT and PCD-CT, using cadaveric human lung specimens and corresponding histological images.

## Materials and methods

### Study Subjects

This retrospective study utilized 20 human cadaveric lungs presenting with nodules or ground-glass opacities, with approval obtained from the institutional review board at Osaka University Hospital. The cadaveric lung specimens, evaluation methods, and visual evaluators used in this study were the same as those in our previous study, which focused solely on PCD-CT [15]. The requirement for informed consent for the use of biomaterials as the materials was waived as it had been obtained previously, and because the data were completely anonymized. Patient records or clinical diagnoses were not available. The lungs were inflated and fixed using the Heitzman method [17].

### CT image acquisition

Conventional EID-CT (CCT; Aquilion ONE; Canon Medical Systems Corp.) and UHR-CT (Aquilion Precision; Canon Medical Systems Corp.) were used in this study. The cadaveric lungs were placed inside plastic cases and scanned. The tube current and volumetric CT dose index were set to 50 mA and 1.7 mGy for CCT, and 40 mA and 1.7 mGy for UHR-CT, respectively. The radiation dose parameters were established according to the preliminary scanning experiment (refer to the supplementary document). The noise levels at 1.7 mGy using the plastic case were comparable to those using a thorax phantom at the diagnostic reference level [18]. UHR-CT scans were conducted in super-high-resolution mode. The collimation settings were 0.5 mm × 80 rows for CCT and 0.25 mm × 160 rows for UHR-CT. Other scan parameters common to both CT systems included a peak potential of 120 kVp, a gantry rotation speed of 0.5 s, and a pitch factor of 0.8. The scan acquisition time for each lung was approximately 6 to 7 s.

PCD-CT imaging was performed as described in our previous study, in which CT images were acquired using PCD-CT (NAEOTOM Alpha, Siemens Healthineers) [15]. A preliminary PCD-CT scan experiment demonstrated that an image acquired at 1.2 mGy (15 mAs) in a plastic case exhibited a noise level comparable to that of an image acquired at the diagnostic reference level in a thorax phantom. Based on this finding, PCD-CT images were acquired at 1.2 mGy (15 mAs). The collimation for PCD-CT was 0.2 mm × 120 rows, and scanning was performed in ultra-high-resolution (UHR) mode. The other scan parameters were as follows: a gantry rotation speed of 0.5 s, a peak potential of 120 kVp, and a pitch factor of 0.8. The acquisition time was approximately 10 s per lung.

Seven image sets were reconstructed with different settings for CT types (CCT, UHR-CT, and PCD-CT), matrix size (512, 1024, and 2048), IR, and DLR: CCT-512-IR, UHR-512-IR, UHR-1024-IR, UHR-2048-IR, UHR-1024-DLR, PCD-512-IR, and PCD-1024-IR. The slice thickness/increment was 0.5 mm/0.5 mm for CCT-512-IR and UHR-512-IR, 0.25 mm/0.25 mm for UHR-1024-IR, UHR-2048-IR, and UHR-1024-DLR, 0.6 mm/0.6 mm for PCD-512-IR, and 0.2 mm/0.2 mm for PCD-1024-IR. The reconstruction settings are summarized in Supplementary Table S1.

All images were reconstructed with a 350 mm field of view. On EID-CTs (CCT and UHR-CT), Adaptive Iterative Dose Reduction 3D (AIDR3D) was employed for IR, while the Advanced Intelligent Clear-IQ Engine (AiCE) was used for DLR. The FC51 reconstruction kernel was used with AIDR3D, whereas the lung setting was applied for AiCE. AIDR3D with an e-Mild setting was employed for CCT-512-IR and UHR-512-IR. However, a preliminary review indicated that AIDR3D with e-Mild for UHR-1024-IR and UHR-2048-IR resulted in excessive visual noise. Therefore, AIDR3D with e-Strong was used for UHR-1024-IR and UHR-2048-IR. The CCT system used in this study had a maximum matrix size of 512 and a minimum slice thickness of 0.5 mm, whereas UHR-CT allowed for a minimum slice thickness of 0.25 mm. The largest available matrix sizes were 2048 for AIDR3D and 1024 for AiCE. For PCD-CT images, a Br64 reconstruction kernel and quantum IR with a strength setting of 3 were used. The PCD-CT system in this study had a maximum matrix size of 1024 and a minimum slice thickness of 0.2 mm.

### Histological images

The CT images were evaluated by the primary investigator (A.H., with 12 years of experience) and slices containing nodules were identified. The lungs were sectioned at a location corresponding to the identified slice, and tissue samples measuring 3 × 2.5 cm² were collected. These samples were stained with hematoxylin and eosin, and virtual histological slides were generated. The primary investigator identified the nodules and airways in the histological images and measured the long-axis diameter of the nodules and the internal diameter of the airways using virtual slide software (NDP.view, version 2.9.29). A pathologist (M.K. with 12 years of experience) reviewed the pathological characteristics of the nodules.

### Visual evaluation

The primary investigator (A.H., with 12 years of experience) examined the CT images and identified the slices corresponding to the histological sections. Two chest radiologists (K.N. with 7 years of experience and M.Y. with 21 years of experience), blinded to the imaging settings, independently compared the identified CT slices with the corresponding histological sections. Nodules were graded on a five-point scale: 1, not identifiable; 2, barely identifiable; 3, clearly identifiable but challenging to assess morphology; 4, clearly identifiable with a nodule shape approximately matching histology; and 5, clearly identifiable with margins and internal texture closely matching histology. Airways were evaluated using a four-point scale: 1, not identifiable; 2, barely identifiable; 3, clearly identifiable, but either the lumen or wall was unclear; and 4, both the lumen and wall were distinctly visualized. Any discrepancies between the two radiologists were resolved by a third radiologist (N.K., with 13 years of experience), who selected one of the scores as the final score.

### Objective noise evaluation

Objective noise for each image set was assessed by measuring the standard deviation (SD) within a region of interest (ROI). Three circular ROIs, each with a diameter of 10 mm, were placed in normal lung regions, avoiding major blood vessels, airways, and lesions. These ROIs were positioned at approximately the same location across all image sets. The signal-to-noise ratio (SNR) for each ROI was calculated using the formula: SNR = |HU_ROI_| /SD_ROI_.

### Statistical analysis

The level of agreement in visual scoring between the two readers was assessed using the weighted kappa coefficient (κw) [19]. The Wilcoxon signed-rank test was employed to evaluate differences in visual scores and objective noise, with Bonferroni correction applied for multiple comparisons. Statistical analyses were conducted for the following nine pairs: CCT-512-IR vs. UHR-512-IR, UHR-512-IR vs. UHR-1024-IR, UHR-1024-IR vs. UHR-2048-IR, UHR-1024-IR vs. UHR-1024-DLR, UHR-2048-IR vs. UHR-1024-DLR, UHR-512-IR vs. PCD-512-IR, UHR-1024-IR vs. PCD-1024-IR, UHR-2048-IR vs. PCD-1024-IR, and UHR-1024-DLR vs. PCD-1024-IR This selection was made to limit the number of comparisons because including all image series pairs would result in an excessive number. Subgroup analyses were conducted for visual scores based on nodule size and type, categorizing the nodules as either solid or sub-solid (non-solid or part-solid). The median nodule size corresponding to each score was compared among the CT series using the Wilcoxon rank-sum test with Bonferroni correction. A *p*-value of < 0.0056 was considered statistically significant following the Bonferroni adjustment. All statistical analyses were conducted using R software version 4.2.2 (R Foundation for Statistical Computing).

## Results

In total, 67 nodules (46 solid and 21 sub-solid) and 92 airways were evaluated in 40 specimens. The size distribution of the nodules and airways is shown in Supplementary Fig. S2. The pathological findings of the nodules are summarized in the Supplementary document. The interobserver agreement for nodules between the two readers was rated as good for CCT and PCD-CT series (CCT-512-IR: κw = 0.740; PCD-512-IR: κw = 0.767; and PCD-1024-IR: κw = 0.783), and excellent for the UHR-CT series (UHR-512-IR: κw = 0.801; UHR-1024-IR: κw = 0.911; UHR-2048-IR: κw = 0.883; UHR-1024-DLR: κw = 0.826). For airways, the agreement was excellent in CCT-512-IR (κw = 0.833), UHR-512-IR (κw = 0.831), and UHR-1024-DLR (κw = 0.878), while it was rated as good in UHR-1024-IR (κw = 0.760), UHR-2048-IR (κw = 0.784), and PCD-CT series (PCD-512-IR: κw = 0.767; PCD-1024-IR: κw = 0.783).

Discrepancies between the two radiologists were identified in 17 nodules (25%) for CCT-512-IR, 23 nodules (34%) for UHR-512-IR, 16 nodules (24%) for UHR-1024-IR, 19 nodules (28%) for UHR-2048-IR, 23 nodules (34%) for UHR-1024-DLR, 27 nodules (40%) for PCD-512-IR, and 27 nodules (40%) for PCD-1024-IR. Discrepancies were noted in 18 airways (20%) for CCT-512-IR, 30 airways (33%) for UHR-512-IR, 34 airways (37%) for UHR-1024-IR, 30 airways (33%) for UHR-2048-IR, 20 airways (22%) for UHR-1024-DLR, 23 airways (25%) for PCD-512-IR, and 33 airways (36%) for PCD-1024-IR.

### Visual scores for nodules

**Fig. 1:**
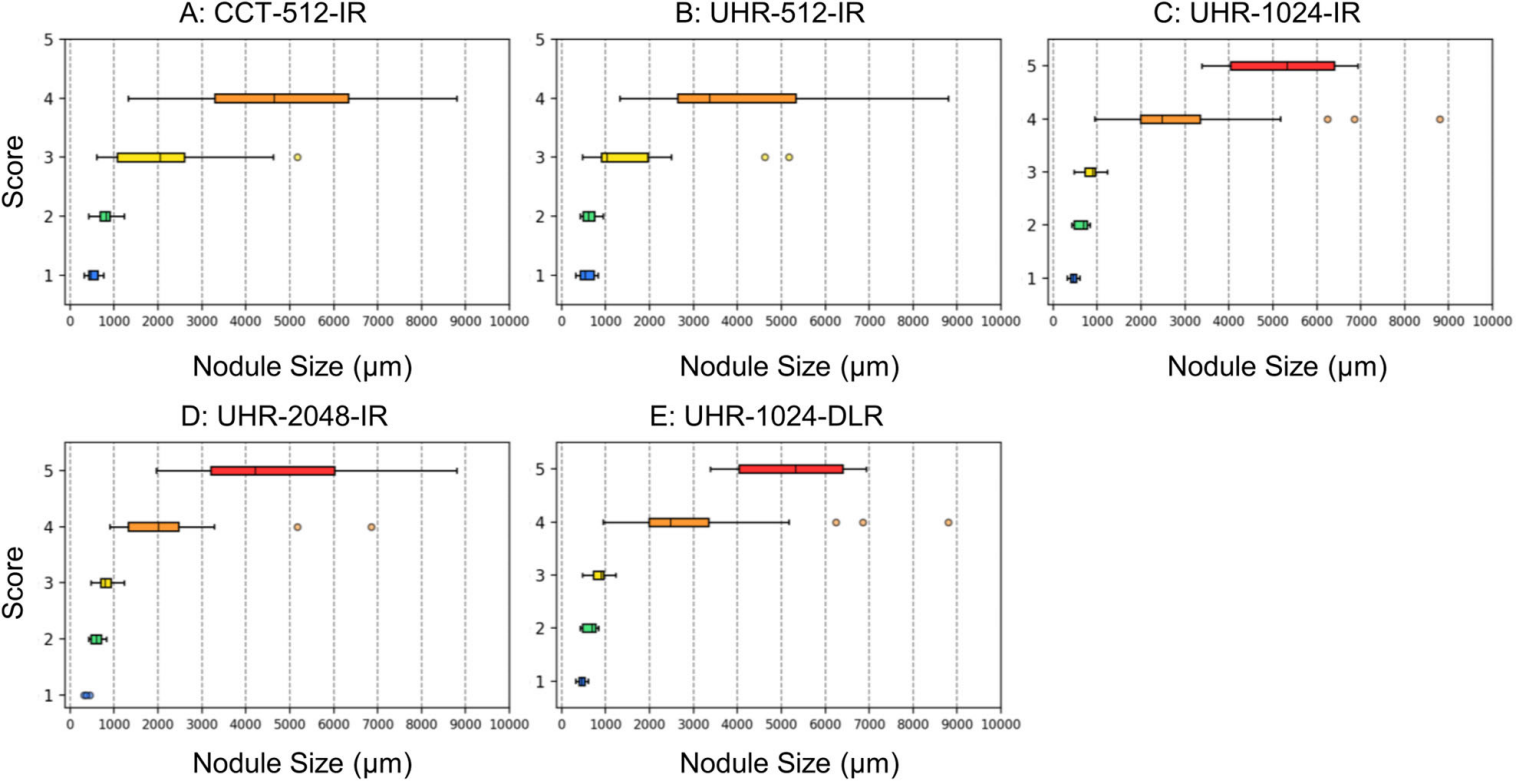
Visual score for nodules and nodule size (box and whisker plots). (**A**) CCT-512-IR, (**B**) UHR-512-IR, (**C**) UHR-1024-IR, (**D**) UHR-2048-IR, and (**E**) UHR-1024-DLR. CCT, conventional CT; UHR, ultra-high-resolution; IR, iterative reconstruction; DLR, deep-learning reconstruction

**Fig. 2:**
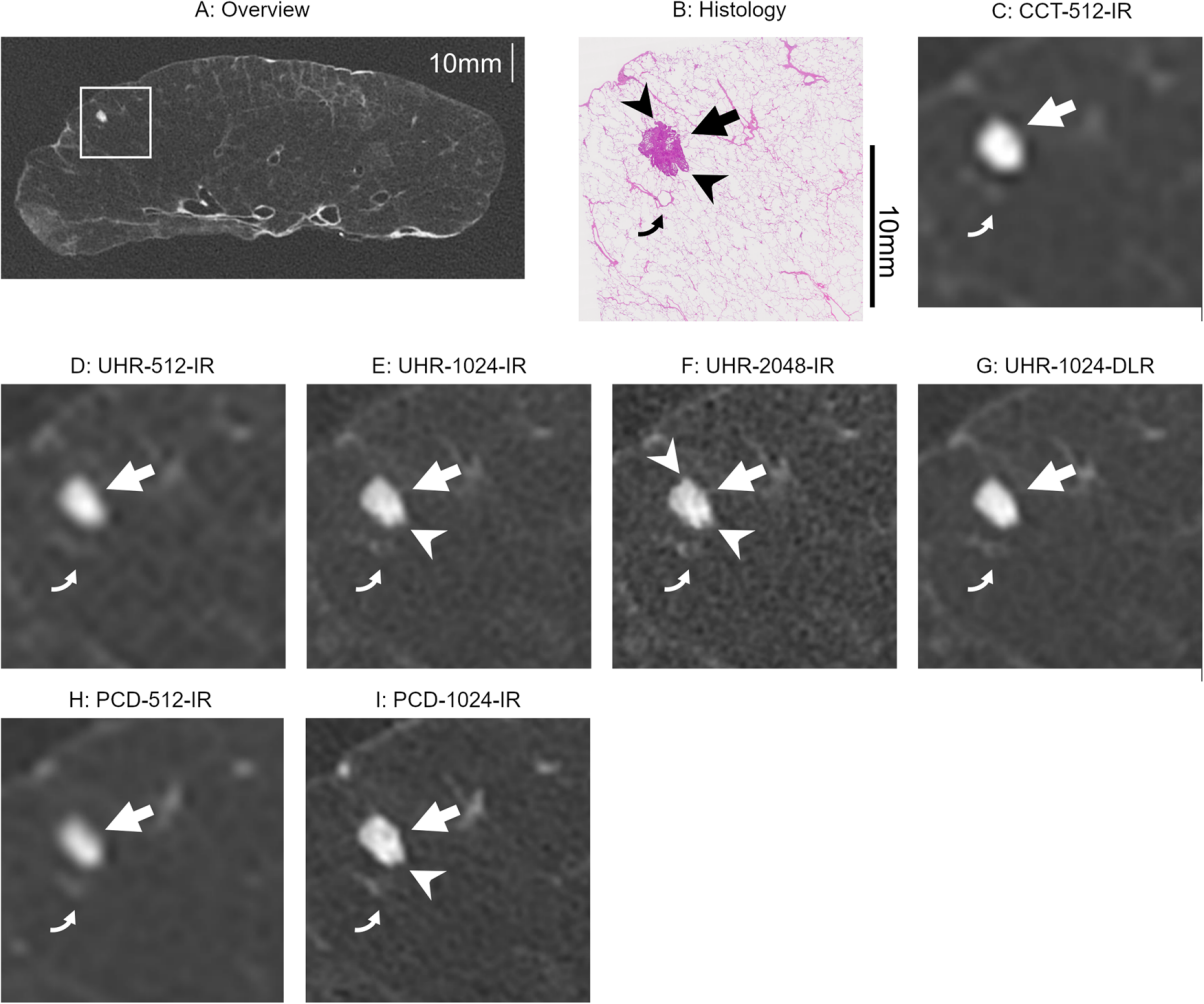
A metastasis of adenocarcinoma (straight arrows; 3385 μm in the long diameter) and a bronchiole (curved arrows; 1125 μm in the inner diameter). **A** Overview of CT image and (**B**) histology. **C** Image with an enlarged local area (square in **A**) on CCT-512-IR, (**D**) UHR-512-IR, (**E**) UHR-1024-IR, (**F**) UHR-2048-IR, (**G**) UHR-1024-DLR, (**H**) PCD-512-IR, and (**I**) PCD-1024-IR. The UHR-2048-IR and PCD-1024-IR depicted the irregular margins (arrowheads). The irregular margins were slightly obscured on the UHR-1024-IR and partly smoothed on the UHR-1024-DLR. Although the nodule margins were obscured, the shape of the nodule is roughly consistent with the histology. On the CCT-512-IR, undershoots were seen around the nodule, and morphological evaluation of the shape and margins is difficult. The lumen and wall of the bronchiole were delineated on the UHR-1024-IR, UHR-2048, and UHR-1024-DLR. The bronchiole was not depicted on the CCT-512-IR, UHR-512-IR, and PCD-512-IR and was obscured on the PCD-1024-IR. CCT, conventional CT; UHR, ultra-high-resolution CT; IR, iterative reconstruction; DLR, deep-learning reconstruction; PCD, photon-counting detector CT

**Fig. 3:**
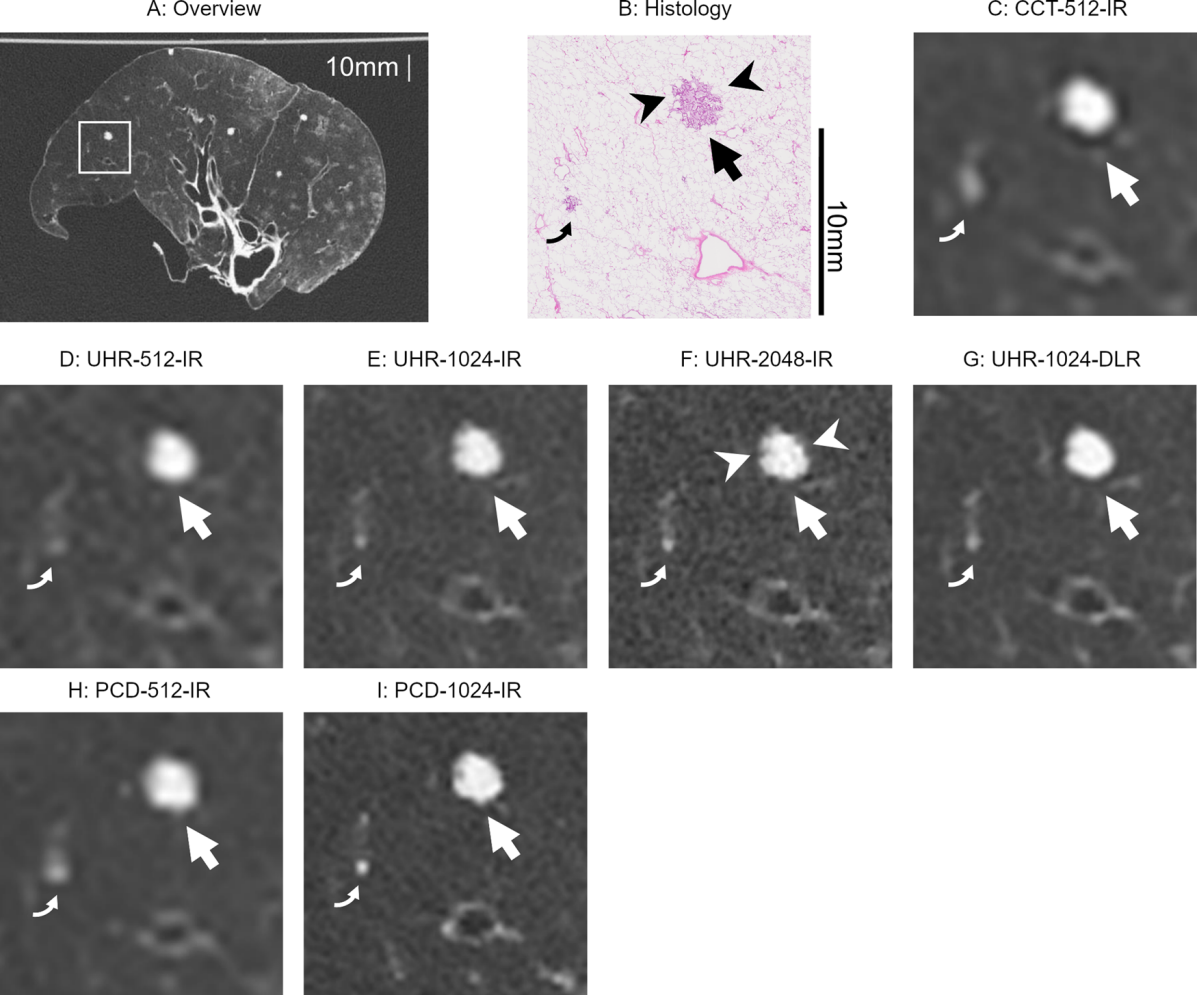
Metastases of adenocarcinoma (straight arrows, 3073 μm; curved arrows, 902 μm). **A** Overview of CT image and (**B**) histology. **C** Image with an enlarged local area (square in **A**) on CCT-512-IR, (**D**) UHR-512-IR, (**E**) UHR-1024-IR, (**F**) UHR-2048-IR, (**G**) UHR-1024-DLR, (**H**) PCD-512-IR, and (**I**) PCD-1024-IR. The UHR-2048-IR clearly delineated the irregular margins of the nodule (arrowheads), measuring 3073 μm in diameter. In comparison, the irregular margins appeared slightly obscured on the UHR-1024-IR and partially smoothed on the UHR-1024-DLR. On the 512 matrix images, the nodule margins were indistinct. The 902 μm nodule (curved arrows) was clearly visualized on the UHR-1024-IR, UHR-2048-IR, UHR-1024-DLR, and PCD-1024-IR but was obscured on the 512 matrix images. CCT, conventional CT; UHR, ultra-high-resolution CT; IR, iterative reconstruction; DLR, deep-learning reconstruction, PCD, photon-counting detector CT

**Fig. 4:**
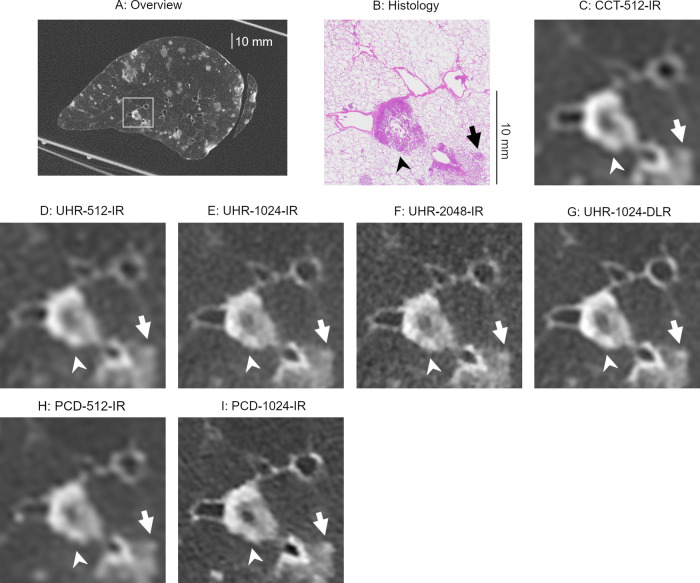
Images showing metastases of adenocarcinoma. **A** Overview of CT image and (**B**) histology. **C** Image with an enlarged local area (square in **A**) on CCT-512-IR, (**D**) UHR-512-IR, (**E**) UHR-1024-IR, (**F**) UHR-2048-IR, (**G**) UHR-1024-DLR, (**H**) PCD-512-IR, and (**I**) PCD-1024-IR. All CT images depicted the nodule with a diameter of 6269 μm (arrowheads). Visual image noise within the nodule is noticeable in UHR-1024-IR and UHR-2048-IR, but not in UHR-1024-DLR. In the 5820 µm nodule, the solid portion (arrows) is most clearly delineated in UHR-1024-DLR. CCT, conventional CT; UHR, ultra-high-resolution CT; IR, iterative reconstruction; DLR, deep-learning reconstruction; PCD, photon-counting detector CT

The visual scores for the nodules are presented in Table 1. Figure 1 displays box-and-whisker plots illustrating the scores and nodule sizes on EID-CTs. The box-and-whisker plots of the PCD-CT scores are shown in our previous paper [15]. Representative images of the nodules are provided in Figs. 2, 3, and 4. The EID-CT images corresponding to the figures in our previous paper [15] are presented in Figs. S2–S6. Among the IR images on EID-CTs, the highest score was achieved by UHR-2048-IR, followed by UHR-1024-IR, UHR-512-IR, and CCT-512-IR, in that order. Statistically significant differences were found between the groups (*p* < 0.001). However, no significant difference was observed between UHR-1024-DLR and UHR-1024-IR (*p* = 0.144) or between UHR-1024-DLR and UHR-2048-IR (*p* = 0.043). No significant difference was observed between UHR-512-IR and PCD-512-IR (*p* = 1.0). PCD-1024-IR scored significantly better than UHR-1024-IR and UHR-1024-DLR (*p* < 0.001). There was no significant difference between UHR-2048-IR and PCD-1024-IR (*p* = 0.041).

**Table 1:** Visual scores for nodules

		Total *N* = 67	Size < 500 μm *N* = 8	Size 500–1000 μm *N* = 19	Size > 1000 μm *N* = 40	Solid *N* = 46	Sub-solid *N* = 21
CCT-512-IR	Score (1/2/3/4/5)	8/17/26/16/0	5/3/0/0/0	3/12/4/0/0	0/2/22/16/0	7/17/17/5/0	8/17/26/16/0
UHR-512-IR	Score (1/2/3/4/5)	8/8/26/25/0	4/3/1/0/0	4/5/10/0/0	0/0/15/25/0	8/7/22/9/0	8/8/26/25/0
UHR-1024-IR	Score (1/2/3/4/5)	6/8/16/30/7	4/3/1/0/0	2/5/11/1/0	0/0/4/29/7	6/8/15/15/2	6/8/16/30/7
UHR-2048-IR	Score (1/2/3/4/5)	3/9/16/21/18	3/3/2/0/0	0/6/11/2/0	0/0/3/19/18	3/9/15/13/6	3/9/16/21/18
UHR-1024-DLR	Score (1/2/3/4/5)	3/9/18/28/9	2/5/1/0/0	1/4/12/2/0	0/0/5/26/9	3/9/16/18/0	3/9/18/28/9
PCD-512-IR	Score (1/2/3/4/5)	9/8/23/26/1	5/1/2/0/0	4/7/8/0/0	0/0/13/26/1	9/7/18/12/0	0/1/5/14/1
PCD-1024-IR	Score (1/2/3/4/5)	6/5/15/13/28	5/1/2/0/0	1/4/11/3/0	0/0/2/10/28	6/5/14/9/12	0/0/1/4/16
*p*-values							
CCT-512-IR vs. UHR-512-IR		< 0.001^a^	0.625	0.180	< 0.001^a^	0.008	0.031
UHR-512-IR vs. UHR-1024-IR		< 0.001^a^	1.000	0.234	< 0.001^a^	0.007	0.002^a^
UHR-1024-IR vs. UHR-2048-IR		< 0.001^a^	0.500	0.125	< 0.001^a^	< 0.001^a^	0.016
UHR-1024-IR vs. UHR-1024-DLR		0.144	0.625	0.125	1.000	0.344	0.453
UHR-2048-IR vs. UHR-1024-DLR		0.043	1.000	1.000	0.003^a^	0.169	0.219
UHR-512-IR vs. PCD-512-IR		1.000	1.000	0.781	0.453	1.000	1.000
UHR-1024-IR vs. PCD-1024-IR		< 0.001^a^	1.000	0.125	< 0.001^a^	< 0.001^a^	< 0.001^a^
UHR-2048-IR vs. PCD-1024-IR		0.041	0.750	1.000	0.001^a^	0.213	0.125
UHR-1024-DLR vs. PCD-1024-IR		< 0.001^a^	0.625	1.000	< 0.001^a^	0.019	0.008

*C-CT* conventional energy-integrating detector CT, *UHR* ultra-high-resolution energy-integrating detector CT, *IR* iterative reconstruction, *DLR* deep-learning reconstruction, *PCD* photon-counting detector CT

^a^ Compared with Wilcoxon signed-rank test with Bonferroni correction. *p-*values < 0.0056 were considered significant

Subgroup analysis based on nodule size revealed no significant differences among the series for nodules smaller than 500 μm or those between 500 μm and 1000 μm in diameter. For nodules larger than 1000 μm, UHR-2048-IR achieved the highest scores among EID-CTs, followed in descending order by UHR-1024-IR, UHR-512-IR, and CCT-512-IR, with statistically significant differences observed (*p* < 0.001). The scores for UHR-2048-IR were significantly higher than those for UHR-1024-DLR (*p* = 0.003). However, no significant difference was observed between the UHR-1024-IR and UHR-1024-DLR groups (*p* = 1.000). PCD-1024-IR scored significantly better than UHR-1024-IR, UHR-2048-IR, and UHR-1024-DLR (*p* ≤ 0.001).

Subgroup analysis of solid nodules showed that UHR-2048-IR achieved significantly higher scores than UHR-1024-IR (*p* < 0.001). No significant difference was observed between the other pairs on EID-CTs (*p* > 0.006). Several solid nodules in UHR-1024-IR and UHR-2048-IR received a score of 5; however, no solid nodules in UHR-1024-DLR received a score of 5. Additionally, the irregular margins of some solid nodules appeared smooth in UHR-1024-DLR (Figs. 2G and 3G). In the analysis of sub-solid nodules, the scores for UHR-512-IR were significantly higher than those for CCT-512-IR (*p* = 0.002). No significant differences were observed among the other pairs on EID-CTs (*p* > 0.01). Several sub-solid nodules in UHR-1024-IR, UHR-2048-IR, and UHR-1024-DLR had a score of 5.

The median sizes of nodules corresponding to scores of 2, 3, 4, or 5 are summarized in Table 2. The median size of barely detectable nodules (score 2) was 696 µm (IQR: 475–772 µm) for UHR-1024-IR, 604 µm (IQR: 469–756 µm) for UHR-2048-IR, 469 µm (IQR: 427–713 µm) for UHR-1024-DLR, 680 µm (IQR: 536–756 µm) for PCD-1024-IR. For nodules with a shape approximately matching histology (score 4), the median size was 2478 µm (IQR: 1983–3348 µm) for UHR-1024-IR, 2014 µm (IQR: 1321–2465 µm) for UHR-2048-IR, 2478 µm (IQR: 1929–3374 µm) for UHR-1024-DLR, and 1232 µm (IQR: 1007–2139 µm) for PCD-1024-IR. PCD-1024-IR showed significantly smaller size for nodules with score 4 than UHR-1024-IR and UHR-1024-DLR. There was no significant difference between UHR-2048-IR and PCD-1024-IR (*p* > 0.1).

**Table 2:** Median size of the nodules with each visual score

		Median size of the nodules with a score of 2 (μm)	Median size of the nodules with a score of 3 (μm)	Median size of the nodules with a score of 4 (μm)	Median size of the nodules with a score of 5 (μm)
CCT-512-IR		819 (676–908)	2047 (1077–2603)	4642 (3296–6331)	N/A
UHR-512-IR		619 (479–744)	1038 (902–1954)	3371 (2641–5329)	N/A
UHR-1024-IR		696 (475–772)	902 (722–956)	2478 (1983–3348)	5329 (4029–6393)
UHR-2048-IR		604 (469–713)	798 (695–925)	2014 (1321–2465)	4217 (3196–6017)
UHR-1024-DLR		469 (427–713)	860 (708–990)	2478 (1929–3374)	5329 (4123–6853)
PCD-512-IR		797 (722, 904)	1026 (759, 2047)	3209 (2221, 4884)	N/A
PCD-1024-IR		680 (536–756)	759 (689, 912)	1232 (1007,2139)	3378 (2484, 5205)
*p-*values					
CCT-512-IR vs. UHR-512-IR		0.179	0.014	0.245	N/A
UHR-512-IR vs. UHR-1024-IR		1.000	0.023	0.026	N/A
UHR-1024-IR vs. UHR-2048-IR		0.593	0.609	0.062	0.250
UHR-1024-IR vs. UHR-1024-DLR		0.382	0.966	0.868	0.979
UHR-2048-IR vs. UHR-1024-DLR		0.529	0.603	0.110	0.425
UHR-512-IR vs. PCD-512-IR		0.152	0.909	0.591	NA
UHR-1024-IR vs. PCD-1024-IR		1.000	0.323	0.002^a^	0.061
UHR-2048-IR vs. PCD-1024-IR		0.640	0.633	0.140	0.229
UHR-1024-DLR vs. PCD-1024-IR		0.369	0.320	0.005^a^	0.127

*C-CT* conventional energy-integrating detector CT, *UHR* ultra-high-resolution energy-integrating detector CT, *IR* iterative reconstruction*, DLR* deep-learning reconstruction, *PCD* photon-counting detector CT

^a^ Compared with the Wilcoxon rank-sum test with Bonferroni correction. Values of *p* < 0.0056 were considered significant

IQR is shown in the parentheses. N/A: The number of nodules with a score of 5 was < 3

### Visual scores for airways

**Fig. 5:**
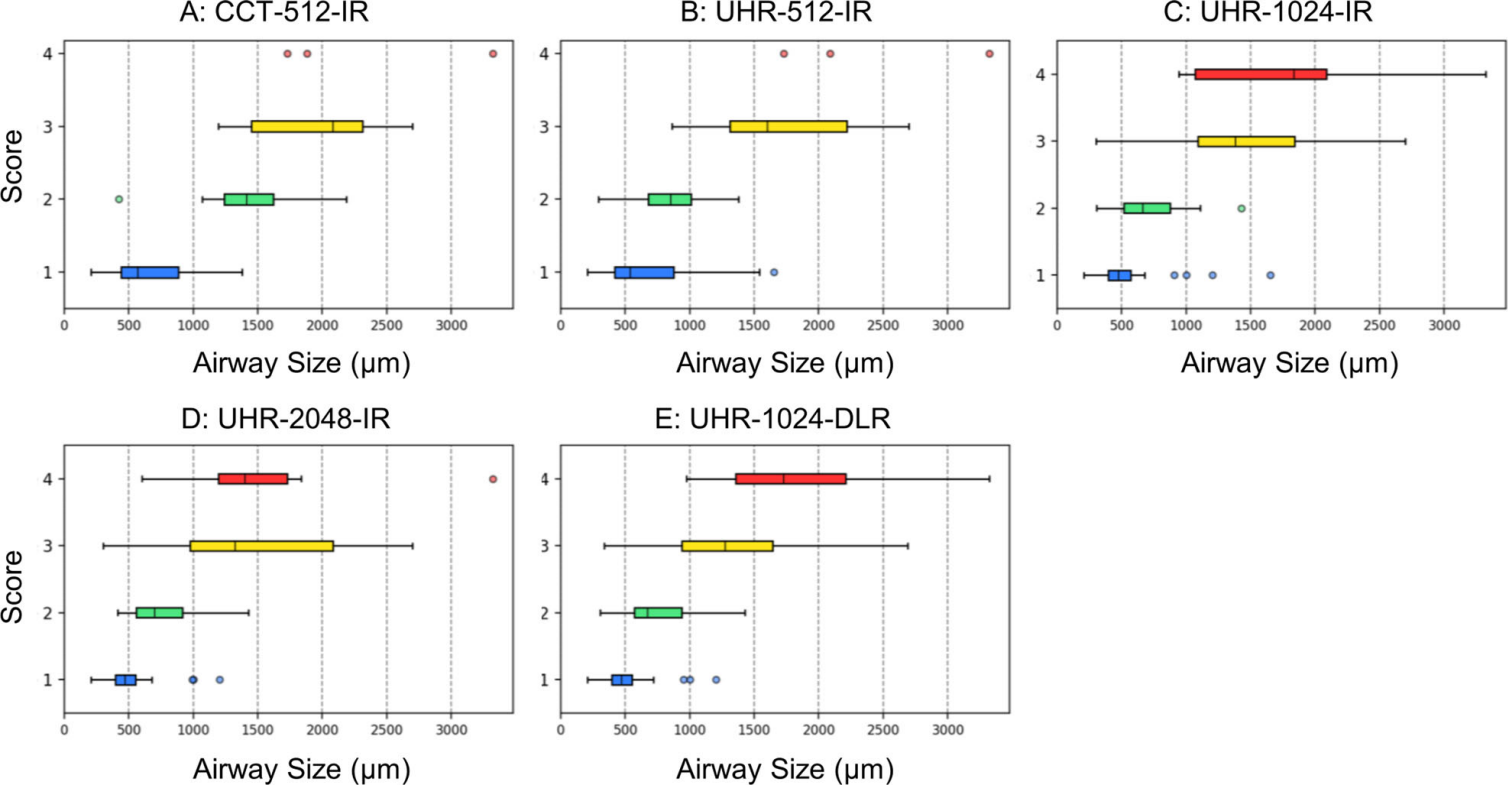
Visual score for airways and airway size (box and whisker plots). (**A**) CCT-512-IR, (**B**) UHR-512-IR, (**C**) UHR-1024-IR, (**D**) UHR-2048-IR, and (**E**) UHR-1024-DLR. CCT, conventional CT; UHR, ultra-high-resolution; IR, iterative reconstruction; DLR, deep-learning reconstruction

**Fig. 6:**
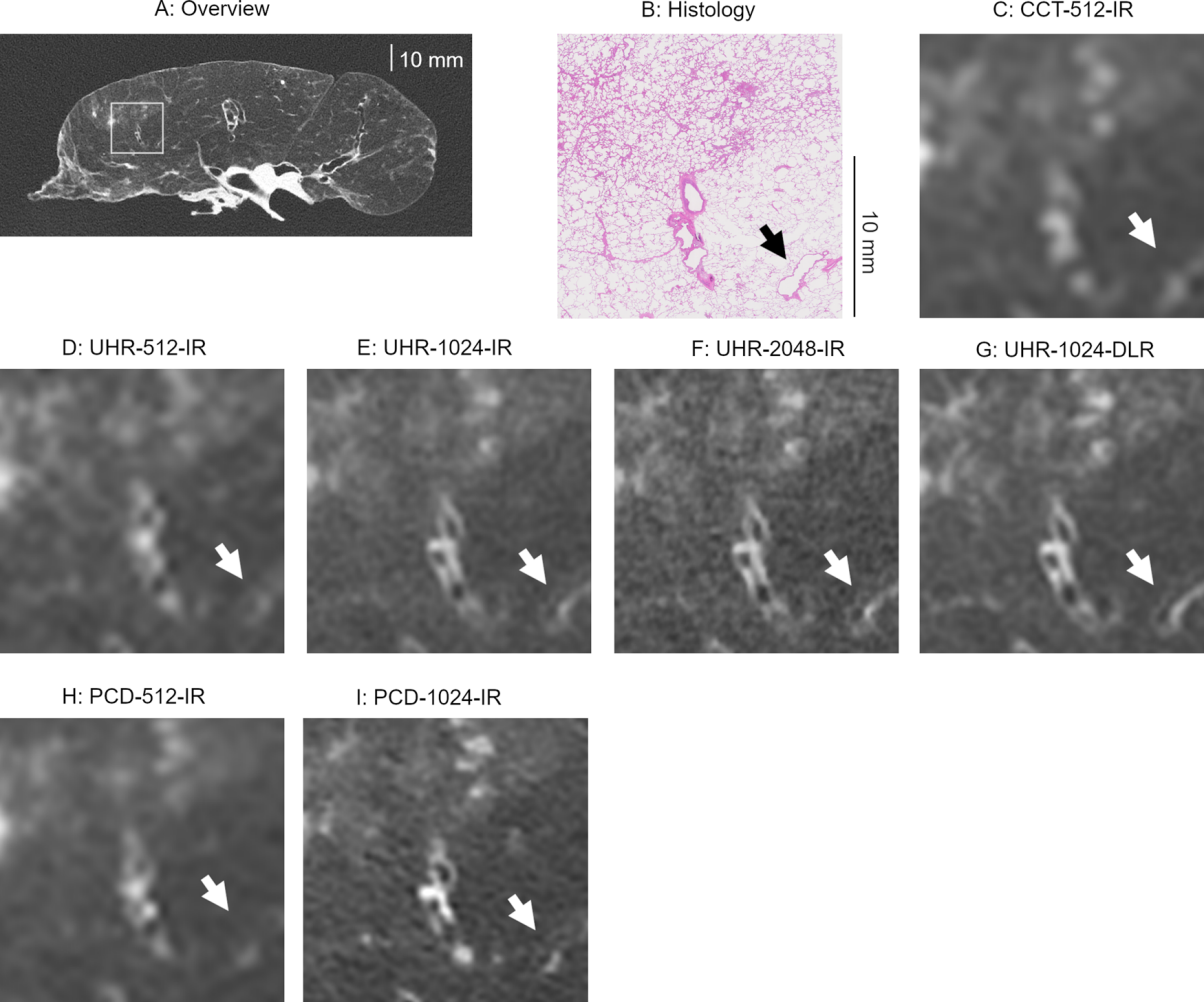
Images showing bronchioles with inner diameters of 855 μm (arrows). **A** Overview of CT image and (**B**) histology. **C** Image with an enlarged local area (square in **A**) on CCT-512-IR, (**D**) UHR-512-IR, (**E**) UHR-1024-IR, (**F**) UHR-2048-IR, (**G**) UHR-1024-DLR, (**H**) PCD-512-IR, and (**I**) PCD-1024-IR. Bronchioles were obscured on the 512 matrix images, but were observed on UHR-1024-IR, UHR-2048-IR, UHR-1024-DLR, and PCD-1024-IR. UHR-1024-DLR showed the bronchioles most clearly. CCT, conventional CT; UHR, ultra-high-resolution CT; IR, iterative reconstruction; DLR, deep-learning reconstruction; PCD, photon-counting detector CT

The visual scores of the airways are presented in Table 3. Figure 5 displays box-and-whisker plots illustrating the scores and airway sizes. Representative images of the airways are provided in Figs. 2 and 6. Although UHR-512-IR showed higher scores than CCT-512-IR, the difference was not statistically significant after Bonferroni correction (*p* = 0.006). UHR-1024-IR outperformed UHR-512-IR (*p* < 0.001). UHR-1024-DLR detected the highest number of airways with a score of 4; however, no significant differences were found between UHR-1024-IR, UHR-2048-IR, and UHR-1024-DLR (*p* > 0.01; Fig. 6E–G). There was no significant difference among the pairs of UHR-CT and PCD-CT (*p* > 0.1).

**Table 3:** Visual scores for airways

		Total *N* = 92	Size < 500 μm *N* = 23	Size 500–1000 μm *N* = 33	Size > 1000 μm *N* = 36
CCT-512-IR	Score (1/2/3/4)	63/11/15/3	22/1/0/0	33/0/0/0	8/10/15/3
UHR-512-IR	Score (1/2/3/4)	55/11/23/3	22/1/0/0	25/7/1/0	8/3/22/3
UHR-1024-IR	Score (1/2/3/4)	33/20/34/5	17/5/1/0	13/13/6/1	3/2/27/4
UHR-2048-IR	Score (1/2/3/4)	30/20/33/9	16/4/3/0	12/13/6/2	2/3/24/7
UHR-1024-DLR	Score (1/2/3/4)	32/21/24/15	17/5/1/0	13/13/6/1	2/3/17/14
PCD-512-IR	Score (1/2/3/4)	55/17/19/1	23/0/0/0	24/9/0/0	8/8/19/1
PCD-1024-IR	Score (1/2/3/4)	28/29/29/6	15/7/1/0	9/18/5/1	4/4/23/5
*p-*values					
CCT-512-IR vs. UHR-512-IR		0.006	1.000	0.008	0.169
UHR-512-IR vs. UHR-1024-IR		< 0.001^a^	0.109	< 0.001^a^	0.009
UHR-1024-IR vs. UHR-2048-IR		0.089	0.375	0.590	0.432
UHR-1024-IR vs. UHR-1024-DLR		0.100	1.000	1.000	0.011
UHR-2048-IR vs. UHR-1024-DLR		1.000	0.500	0.633	0.065
UHR-512-IR vs. PCD-512-IR		0.206	1.000	1.000	0.222
UHR-1024-IR vs. PCD-1024-IR		0.864	0.688	0.629	0.549
UHR-2048-IR vs. PCD-1024-IR		0.324	0.972	1.000	0.157
UHR-1024-DLR vs. PCD-1024-IR		0.243	0.789	0.680	0.005^a^

*C-CT* conventional energy-integrating detector CT, *UHR* ultra-high-resolution energy-integrating detector CT, *IR* iterative reconstruction, *DLR* deep-learning reconstruction, *PCD* photon-counting detector CT

^a^ Compared with Wilcoxon signed-rank test with Bonferroni correction. Values of *p* < 0.0056 were considered significant

Subgroup analysis based on airway size showed that the scores for UHR-1024-IR were significantly higher than those for UHR-512-IR for airways with diameters of 500 μm–1000 μm (*p* < 0.001). No significant differences were observed among the other comparisons on EID-CTs (*p* > 0.01). For airways with diameters > 1000 μm, UHR-1024-DLR showed significantly higher scores than PCD-1024-IR (*p* = 0.005).

The median sizes of airways with scores of 2, 3, and 4 are summarized in Table 4. A significant difference was observed between UHR-512-IR and CCT-512-IR for airways with a score of 2 (*p* = 0.002), while no significant differences were found among the other comparisons (*p* > 0.1). The median size of barely detectable airways (score 2) was 665 µm (IQR: 516–874 µm) for UHR-1024-IR, 699 µm (IQR: 559–918 µm) for UHR-2048-IR, 675 µm (IQR: 571–937 µm) for UHR-1024-DLR, and 601 µm (IQR: 520–906 µm) for PCD-1024-IR.

**Table 4:** Median size of the airways with each visual score

	Median size of the airways with a score of 2 (μm)	Median size of the airways with a score of 3 (μm)	Median size of the airways with a score of 4 (μm)
CCT-512-IR	1412 (1238–1623)	2083 (1450–2314)	1880 (1804–2602)
UHR-512-IR	855 (677–1009)	1605 (1313–2217)	2086 (1907–2705)
UHR-1024-IR	665 (516–874)	1383 (1089–1842)	1836 (1068–2086)
UHR-2048-IR	699 (559–918)	1324 (974–2083)	1399 (1195–1728)
UHR-1024-DLR	675 (571–937)	1273 (937–1644)	1728 (1355–2210)
PCD-512-IR	942 (849, 1412)	1501 (1288, 2166)	N/A
PCD-1024-IR	601 (520–906)	1324 (1068–1605)	1858 (1755–2220)
*p*-values			
CCT-512-IR vs. UHR-512-IR	0.002^a^	0.276	1.000
UHR-512-IR vs. UHR-1024-IR	0.186	0.110	0.536
UHR-1024-IR vs. UHR-2048-IR	0.625	0.748	0.540
UHR-1024-IR vs. UHR-1024-DLR	0.821	0.440	0.851
UHR-2048-IR vs. UHR-1024-DLR	0.842	0.709	0.256
UHR-512-IR vs. PCD-512-IR	0.137	0.876	0.750
UHR-1024-IR vs. PCD-1024-IR	0.691	0.694	0.825
UHR-2048-IR vs. PCD-1024-IR	0.394	0.992	0.140
UHR-1024-DLR vs. PCD-1024-IR	0.523	0.726	0.635

*C-CT* conventional energy-integrating detector CT, *UHR* ultra-high-resolution energy-integrating detector CT, *IR* iterative reconstruction, *DLR* deep-learning reconstruction, *PCD* photon-counting detector CT

IQR is shown in the parentheses. *N/A*: The number of airways with a score of 4 was < 3

^a^ Compared with Wilcoxon rank-sum test with Bonferroni correction. Values of *p* < 0.0056 were considered significant

### Objective noise

The objective noise and SNR values are presented in Table 5. Among EID-CTs, CCT-512-IR exhibited the lowest noise, followed by UHR-1024-DLR, UHR-1024-IR, UHR-512-IR, and UHR-2048-IR, in ascending order, with statistically significant differences (all *p* < 0.0056). CCT-512-IR exhibited the highest SNR, followed by UHR-1024-DLR, UHR-1024-IR, UHR-512-IR, and UHR-2048-IR, in descending order, with statistically significant differences (all *p* < 0.001). PCD-512-IR showed lower noise and higher SNR than UHR-512-IR (*p* < 0.001). PCD-1024-IR showed higher noise and lower SNR than UHR-1024-IR and UHR-1024-DLR (*p* < 0.001) and lower noise and higher SNR than UHR-2048-IR (*p* < 0.001).

**Table 5:** Objective noise measurements

	SD	SNR
CCT-512-IR	23.52 (21.46, 27.54)	38.01 (32.48, 42.14)
UHR-512-IR	43.94 (40.70, 47.38)	20.36 (18.84, 22.84)
UHR-1024-IR	37.20 (35.23, 40.15)	23.95 (22.28, 26.23)
UHR-2048-IR	51.99 (48.96, 55.29)	17.56 (16.14, 18.84)
UHR-1024-DLR	35.49 (31.86, 40.53)	25.33 (21.46, 29.45)
PCD-512-IR	27.50 (23.75, 32.65)	34.23 (27.28, 38.72)
PCD-1024-IR	45.25 (40.33, 51.27)	20.87 (17.46, 23.26)
*p-*values		
CCT-512-IR vs. UHR-512-IR	< 0.001^a^	< 0.001^a^
UHR-512-IR vs. UHR-1024-IR	< 0.001^a^	< 0.001^a^
UHR-1024-IR vs. UHR-2048-IR	< 0.001^a^	< 0.001^a^
UHR-1024-IR vs. UHR-1024-DLR	0.005^a^	< 0.001^a^
UHR-2048-IR vs. UHR-1024-DLR	< 0.001^a^	< 0.001^a^
UHR-512-IR vs. PCD-512-IR	< 0.001^a^	< 0.001^a^
UHR-1024-IR vs. PCD-1024-IR	< 0.001^a^	< 0.001^a^
UHR-2048-IR vs. PCD-1024-IR	< 0.001^a^	< 0.001^a^
UHR-1024-DLR vs. PCD-1024-IR	< 0.001^a^	< 0.001^a^

*SD* standard deviation, *SNR* signal-to-noise ratio, *C-CT* conventional energy-integrating detector CT, *UHR* ultra-high-resolution energy-integrating detector CT, *IR* iterative reconstruction, *DLR* deep-learning reconstruction, *PCD* photon-counting detector CT

The data is shown as median (IQR)

^a^ Compared with Wilcoxon signed-rank test with Bonferroni correction. *p*-values < 0.0056 were considered significant

## Discussion

This study evaluated the depiction capability of fine lung nodules and airways using high-resolution imaging settings on UHR-EID-CT. The findings demonstrated that UHR-CT provided superior delineation of small nodules compared to CCT-512-IR, using histological images as the reference standard under identical reconstruction conditions. Among the UHR-CT images reconstructed using IR, those with a 2048 matrix were significantly better than those with a 1024 matrix. UHR-2048-IR outperformed UHR-1024-DLR in detecting nodules > 1000 μm. For airways, no significant differences were observed among UHR-1024-IR, UHR-2048-IR, and UHR-1024-DLR. UHR-CT showed the potential to detect nodules measuring 0.47–0.70 mm in diameter and airways with diameters of 0.67–0.70 mm. In the comparison between UHR-CT and PCD-CT, no significant difference was observed in images with the 512 matrix (*p* > 0.1). PCD-CT with 1024 matrix outperformed UHR-CTs with high-resolution settings for nodules > 1000 μm (*p* < 0.001), while UHR-1024-DLR scored significantly better than PCD-CT for airways > 1000 μm (*p* = 0.005).

We previously compared UHR-CT and CCT using cadaveric lungs and found that UHR-CT provided superior visualization of normal anatomical structures and abnormal findings [1]. We also reported that larger matrix sizes result in enhanced image quality [2], consistent with the current findings for nodule detection. Our previous report on UHR-CT was based on visual analysis without reliable reference standard images. Consequently, in this study, we evaluated UHR-CT, including the detection of tiny nodules, using histological images as references to further validate the improved image quality. Additionally, this study also included an evaluation of individual nodule sizes, demonstrating the ability to detect small nodules ranging from 0.47 to 0.70 mm in size. However, the clinical significance of detecting nodules smaller than 1 mm remains unclear. This study aimed to evaluate the depiction capability of high-resolution imaging, including the assessment of submillimeter nodules. The potential importance may lie not in the detection of tiny nodules but in the diagnosis facilitated by the margin evaluation of small nodules. Furthermore, Ninomiya et al conducted a radiomics analysis using 1024-matrix images on UHR-EID-CT and reported its utility in predicting solid and micropapillary components of lung adenocarcinoma [20]. High-resolution imaging may influence quantitative assessments such as radiomics and AI-based evaluations of nodules. Further investigation is warranted to clarify its impact.

Regarding improvements in airway visualization with UHR-CT, Morita et al reported that UHR-CT (1024 matrix with 0.25-mm slice thickness) allowed for the segmentation of more distal bronchi compared to the conventional mode (512 matrix with 0.5-mm slice thickness) in automated bronchial segmentation [21]. Similarly, our findings indicate that UHR-1024-IR outperformed UHR-512-IR, consistent with the results reported by Morita et al. Furthermore, UHR-CT was capable of detecting airways as small as 0.67–0.70 mm in diameter. Enhanced detection of airways may lead to more accurate evaluation of airway pathologies and facilitate improved planning for pre-bronchoscopy routes.

Although no significant differences were observed between the IR and DLR images, individual differences were observed in the appearance of the nodules and airways. Some irregular margins on solid nodules appeared smoother, indicating that DLR might generate images with artificially altered characteristics at the microscopic level. Conversely, some airways appeared clearer using DLR. Thus, deep learning may facilitate more accurate predictions for normal or simple structures than for abnormal ones; however, further investigation is required.

Additionally, no differences were observed in the images with the 512 matrix between UHR-CT and PCD-CT. This suggests that there may be minimal differences between PCD-CT and UHR-CT for the matrix size commonly used in clinical practice. PCD-CT was superior to UHR-CT in nodule evaluation using the 1024 matrix images, likely due to the higher spatial resolution of PCD-CT (0.11 mm) compared to that of UHR-CT (0.14 mm). Additionally, UHR-1024-DLR scored better than PCD-1024-IR in the visualization of airways > 1000 μm. The noise-reduction capabilities of DLR may have contributed positively because the PCD-CT images were reconstructed with IR. However, the results may differ if DLR is available for PCD-CT. Nonetheless, it should be noted that the slice thickness (0.25 mm for UHR-CT vs. 0.2 mm for PCD-CT) was not standardized, and each vendor’s unique reconstruction algorithms make direct comparisons challenging. Additionally, objective noise analysis suggested a tendency for higher noise in PCD-CT with 1024 matrix images, which, despite the typical noise-reduction advantage of PCD-CT, implies that the settings used were optimized for high-resolution imaging. These factors complicate precise comparisons.

Despite the positive findings, this study has some limitations that must be considered. First, the use of cadaveric lungs limited our ability to assess factors such as motion artifacts, chest wall influence, and body composition. Thus, in vivo images may be less clear than our results. Second, the primary investigator identified the CT images that matched the histological findings, and only these specific slices were reviewed by the evaluators, not all the slices that were available. This approach was necessary because histological analysis does not always capture the maximum cross-section of the nodules or airways, making multi-slice detection uncertain. Additionally, slight mismatches may have influenced our findings despite careful slice selection. Moreover, coronal and sagittal images were not assessed, and continuity across airway slices was not confirmed. Third, we did not evaluate the impact of different reconstruction settings, such as field of view, reconstruction kernel, and IR and DLR settings, on CT images. In this study, UHR-CT images with a 512-matrix and DLR were not utilized. Given the large number of image sets analyzed, priority was given to images with higher resolution settings. Fourth, the 2048 matrix size results in a data volume that is 16 times larger compared to a 512 matrix. Additionally, 0.25 mm slice thickness generates twice the data volume compared to 0.5 mm slice thickness. Consequently, the UHR-2048-IR images used in this study involve an enormous data volume, which may pose limitations for clinical application. Reducing the field of view can enhance image quality [22], making it a more clinically feasible approach compared to using a large matrix size. Finally, the small sample size limits the statistical power of the study.

In conclusion, the UHR-2048-IR setting achieved the highest scores among the evaluated EID-CT images. UHR-CT demonstrated its potential in detecting submillimeter pulmonary nodules and airways, suggesting improved capability for assessing nodules and airway lesions. Additionally, when utilizing a 512 matrix, UHR-CT exhibited performance comparable to that of PCD-CT.

## Supplementary information

ELECTRONIC SUPPLEMENTARY MATERIAL

## Abbreviations

AiCE: Advanced intelligent clear-IQ Engine
AIDR3D: Adaptive iterative dose reduction 3D
CCT: Conventional CT
DLR: Deep-learning reconstruction
EID-CT: Energy-integrating detector CT
IR: Iterative reconstruction
PCD-CT: Photon-counting detector-CT
ROI: Region of interest
SD: Standard deviation
SNR: Signal-to-noise ratio
UHR: Ultra-high-resolution
UHR-CT: Ultra-high-resolution energy-integrating detector CT

## References

[1] Yanagawa M, Hata A, Honda O et al (2018) Subjective and objective comparisons of image quality between ultra-high-resolution CT and conventional area detector CT in phantoms and cadaveric human lungs. Eur Radiol 28:5060–5068. 10.1007/s00330-018-5491-2 10.1007/s00330-018-5491-2 PMC6223853 29845337

[2] Hata A, Yanagawa M, Honda O et al (2018) Effect of matrix size on the image quality of ultra-high-resolution CT of the lung: comparison of 512 × 512, 1024 × 1024, and 2048 × 2048. Acad Radiol 25:869–876. 10.1016/J.ACRA.2017.11.017 29373211 10.1016/j.acra.2017.11.017

[3] Yanagawa M, Tomiyama N, Honda O et al (2010) Multidetector CT of the lung: image quality with garnet-based detectors. Radiology 255:944–954. 10.1148/radiol.10091010 20501732 10.1148/radiol.10091010

[4] Tsukagoshi S, Ota T, Fujii M et al (2007) Improvement of spatial resolution in the longitudinal direction for isotropic imaging in helical CT. Phys Med Biol 52:791–801. 10.1088/0031-9155/52/3/018 17228121 10.1088/0031-9155/52/3/018

[5] Yanagawa M, Tsubamoto M, Satoh Y et al (2020) Lung adenocarcinoma at CT with 0.25-mm section thickness and a 2048 matrix: high-spatial-resolution imaging for predicting invasiveness. Radiology 297:462–471. 10.1148/radiol.2020201911 32897161 10.1148/radiol.2020201911

[6] Hino T, Kamitani T, Sagiyama K et al (2020) Detectability of the artery of Adamkiewicz on computed tomography angiography of the aorta by using ultra-high-resolution computed tomography. Jpn J Radiol 38:658–665. 10.1007/s11604-020-00943-3 32170567 10.1007/s11604-020-00943-3

[7] Fujiwara M, Watanabe Y, Kashiwagi N et al (2021) Improved visualization of the chorda tympani nerve using ultra-high-resolution computed tomography. Acta Radio Open 10:20584601211061444. 10.1177/20584601211061444 10.1177/20584601211061444 PMC8637724 34868664

[8] Lell MM, Kachelrieß M (2020) Recent and upcoming technological developments in computed tomography: high speed, low dose, deep learning, multienergy. Invest Radiol 55:8–19. 10.1097/RLI.0000000000000601 31567618 10.1097/RLI.0000000000000601

[9] Alkadhi H, Euler A (2020) The future of computed tomography: personalized, functional, and precise. Invest Radiol 55:545–555. 10.1097/RLI.0000000000000668 32209817 10.1097/RLI.0000000000000668

[10] Singh R, Digumarthy SR, Muse VV et al (2020) Image quality and lesion detection on deep learning reconstruction and iterative reconstruction of submillisievert chest and abdominal CT. AJR Am J Roentgenol 214:566–573. 10.2214/AJR.19.21809 31967501 10.2214/AJR.19.21809

[11] McCollough CH, Rajendran K, Leng S et al (2023) The technical development of photon counting detector CT. Eur Radiol 33:5321–5330. 10.1007/s00330-023-09545-9 37014409 10.1007/s00330-023-09545-9 PMC10330290

[12] McCollough CH, Rajendran K, Baffour FI et al (2023) Clinical applications of photon counting detector CT. Eur Radiol 33:5309–5320. 10.1007/s00330-023-09596-y 37020069 10.1007/s00330-023-09596-y PMC10330165

[13] Fletcher JG, Inoue A, Bratt A et al (2024) Photon-counting CT in thoracic imaging: early clinical evidence and incorporation into clinical practice. Radiology 310:e231986. 10.1148/radiol.231986 38501953 10.1148/radiol.231986

[14] Mergen V, Sartoretti T, Baer-Beck M et al (2022) Ultra-high-resolution coronary CT angiography with photon-counting detector CT: feasibility and image characterization. Invest Radiol 57:780–788. 10.1097/RLI.0000000000000897 35640019 10.1097/RLI.0000000000000897 PMC10184822

[15] Hata A, Yanagawa M, Ninomiya K et al (2025) Photon-counting detector CT radiological-histological correlation in cadaveric human lung nodules and airways. Invest Radiol 60:151–160. 10.1097/RLI.0000000000001117 10.1097/RLI.0000000000001117 39159364

[16] Zhou Z, Zhan P, Jin J et al (2017) The imaging of small pulmonary nodules. Transl Lung Cancer Res 6:62–67. 10.21037/tlcr.2017.02.02 28331825 10.21037/tlcr.2017.02.02 PMC5344844

[17] Markarian B (1975) A simple method of inflation–fixation and air drying of lungs. Am J Clin Pathol 63:20–24. 10.1093/ajcp/63.3.20 1089357 10.1093/ajcp/63.3.20

[18] Kanal KM, Butler PF, Sengupta D et al (2017) U.S. diagnostic reference levels and achievable doses for 10 adult CT examinations. Radiology 284:120–133. 10.1148/radiol.2017161911 28221093 10.1148/radiol.2017161911

[19] Landis JR, Koch GG (1977) The measurement of observer agreement for categorical data. Biometrics 33:159–174. 10.2307/2529310 843571

[20] Ninomiya K, Yanagawa M, Tsubamoto M et al (2024) Prediction of solid and micropapillary components in lung invasive adenocarcinoma: radiomics analysis from high-spatial-resolution CT data with 1024 matrix. Jpn J Radiol 42:590–598. 10.1007/s11604-024-01534-2 38413550 10.1007/s11604-024-01534-2 PMC11139717

[21] Morita Y, Yamashiro T, Tsuchiya N et al (2020) Automatic bronchial segmentation on ultra-HRCT scans: advantage of the 1024-matrix size with 0.25-mm slice thickness reconstruction. Jpn J Radiol 38:953–959. 10.1007/s11604-020-01000-9 32562178 10.1007/s11604-020-01000-9

[22] Miyata T, Yanagawa M, Hata A et al (2020) Influence of field of view size on image quality: ultra-high-resolution CT vs. conventional high-resolution CT. Eur Radiol 30:3324–3333. 10.1007/s00330-020-06704-0 32072253 10.1007/s00330-020-06704-0 PMC7248011

