# Supplementary material for: Morphology-based radiological-histological correlation on ultra-high-resolution energy-integrating detector CT using cadaveric human lungs: nodule and airway analysis
Source: Eur Radiol. 2025 Jun 26;35(12):8176–90. doi: 10.1007/s00330-025-11756-1 (PMC12634759; doi:10.1007/s00330-025-11756-1)
Supplement: Supplementary file 1 — ELECTRONIC SUPPLEMENTARY MATERIAL [file 330_2025_11756_MOESM1_ESM.pdf]

Morphology-Based Radiological-Histological Correlation on Ultra-High-Resolution Energy-Integrating Detector CT Using Cadaveric Human Lungs: Nodule and Airway Analysis

ELECTRONIC SUPPLEMENTARY MATERIAL

Supplementary document

Preliminary Scan Experiment

We scanned the lung in a plastic case to obtain CT images in identical cross-sectional directions across the two CT scanners and to facilitate tissue sampling site identification. However, the noise levels within the chest wall differed from clinical scans. Therefore, a preliminary experiment was performed to identify the scan settings matching the noise levels when the cadaveric lung was placed in a thoracic phantom. Consequently, subsequent scans were performed at the diagnostic reference level (DRL) (1).

A cadaveric lung placed in a thorax phantom (N-1 "LUNGMAN," Kyoto Kagaku Co.) was scanned at CTDIvol of 9.1 mGy (210 mA) on UHR-CT (Aquilion Precision; Canon Medical Systems Corp.). The same lung in the plastic case was scanned at tube currents ranging from 10 – 210 mA. Other parameters included the FC51 kernel, 512 matrix, 0.5-mm slice thickness, and 350-mm field of view. Three 1-cm ROIs were placed in the normal lung area to measure the SD values.

The SD values are presented in Supplementary FigureS1. The image at 1.7 mGy (40 mA) in the plastic case had noise nearly equivalent to the 9.1 mGy (210 mA) image in the thorax phantom.

Pathological findings of nodules

The pathological findings of the nodules were as follows: metastasis of malignant tumors (n=47), degenerated red blood cells and inflammatory cells (n=10), fibrosis with carbon deposition (n=4), degenerated red blood cell (n=3), fibrous thickening of the alveolar septa with alveolar hemorrhage (n=2), and degenerated red blood cell with micro abscess (n=1).

References

1. Kanal KM, Butler PF, Sengupta D, et al. U.S. Diagnostic Reference Levels and Achievable Doses for 10 Adult CT Examinations. Radiology. 2017;284:120–133.

2. Hata A, Yanagawa M, Ninomiya K, et al (2024) Photon-Counting Detector CT Radiological-Histological Correlation in Cadaveric Human Lung Nodules and Airways. Invest Radiol Online ahead of print.: https://doi.org/10.1097/RLI.0000000000001117

Supplementary Figure Legends

Figure S1. Graph of the noise in the images of a plastic case scanned with varying tube current.

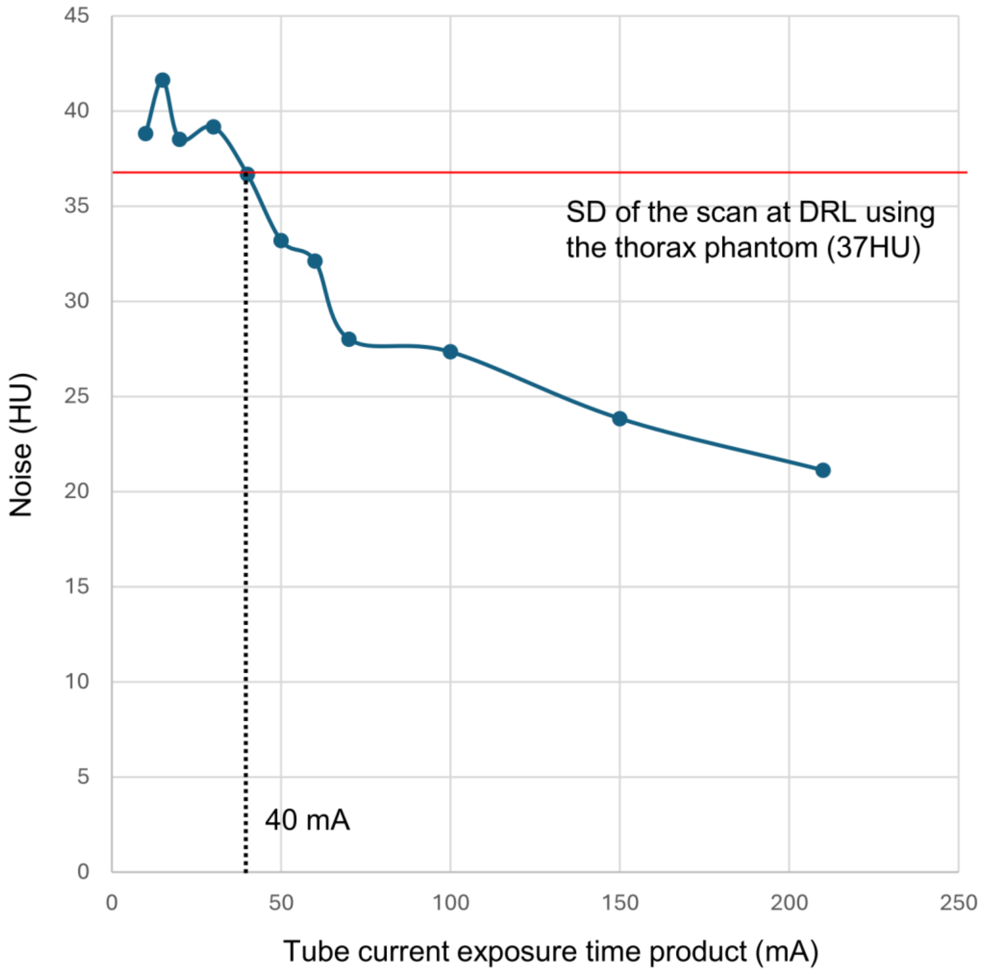

Figure S2. Size distribution of the nodules and airways.

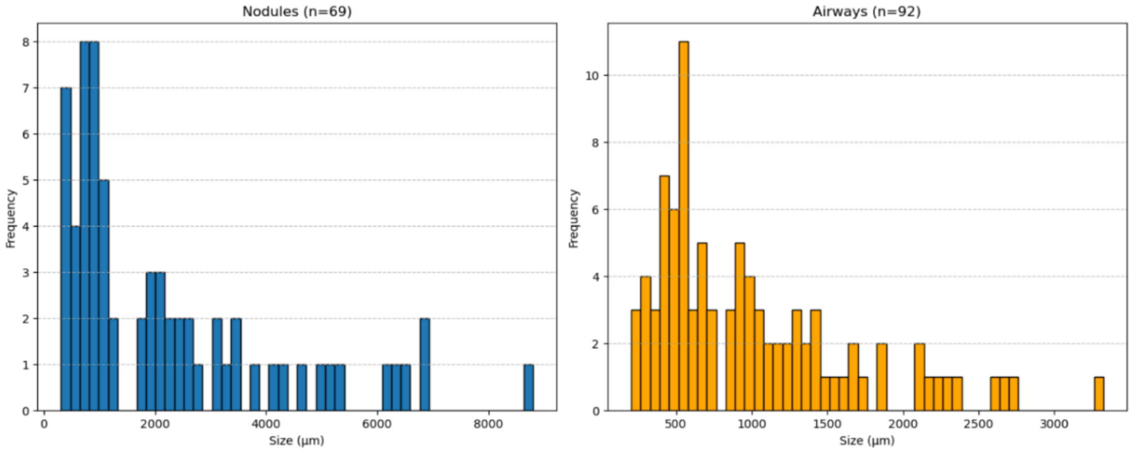

Figure S3: Images of a metastasis of adenocarcinoma with a diameter of 6517 μm (arrows).

(A) Overview of CT image. (B) Image with an enlarged local area (square in A) on CCT-512-IR, (C) UHR-512-IR, (D) UHR-1024-IR, (E) UHR-2048-IR, and (F) UHR-1024-DLR. The UHR-1024-IR and UHR-2048-IR images depicted the nodule shape and irregular margins. The margin of the nodule in the UHR-1024-DLR was partly smoothed (arrowhead).

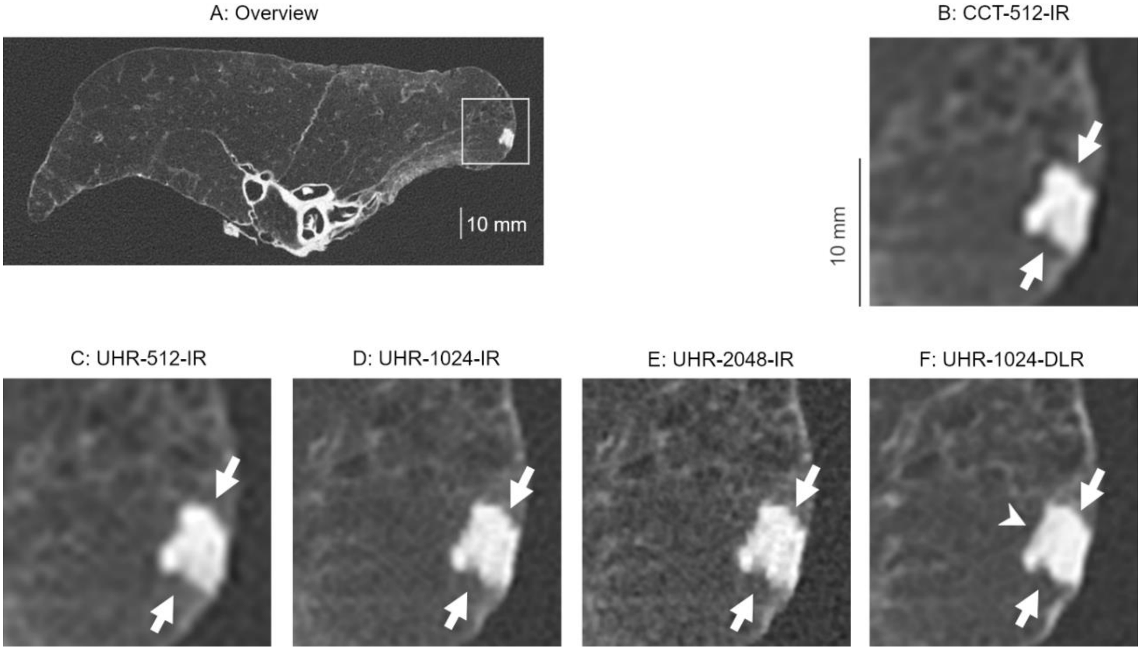

CCT, conventional CT; UHR, ultra-high-resolution CT; IR, iterative reconstruction; DLR, deep learning reconstruction.

The histological image corresponding to the CT images was published in the previous paper (2).

Figure S4: Images showing metastases of adenocarcinoma.

(A) Overview of CT image. (B) Image with an enlarged local area (square in A) on CCT-512-IR, (C) UHR-512-IR, (D) UHR-1024-IR, (E) UHR-2048-IR, and (F) UHR-1024-DLR. Nodules with a diameter of 2139 μm (arrows) are depicted in all images. Nodules with a diameter of 414 μm (arrowheads) are not shown on CCT-512-IR but are shown on UHR-CTs. UHR-2048-IR clearly depicts these nodules.

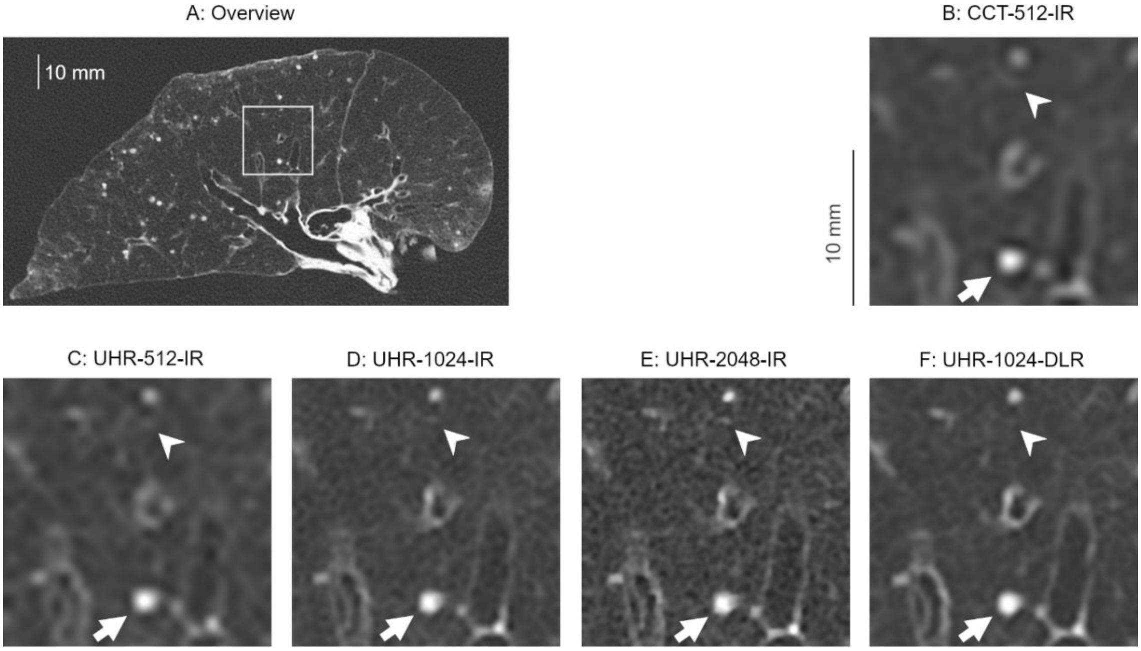

CCT, conventional CT; UHR, ultra-high-resolution CT; IR, iterative reconstruction; DLR, deep learning reconstruction.

The histological image corresponding to the CT images was published in the previous paper (2).

Figure S5: Images showing a bronchiole with a diameter of 1125 μm (arrows). (A) Overview of CT image. (B) Image with an enlarged local area (square in A) on CCT-512-IR, (C) UHR-512-IR, (D) UHR-1024-IR, (E) UHR-2048-IR, and (F) UHR-1024-DLR. The bronchioles were obscured using CCT-512-IR and UHR-512-IR. However, UHR-1024-IR, UHR-2048-IR, and UHR-1024-DLR showed the lumen and walls of the bronchiole.

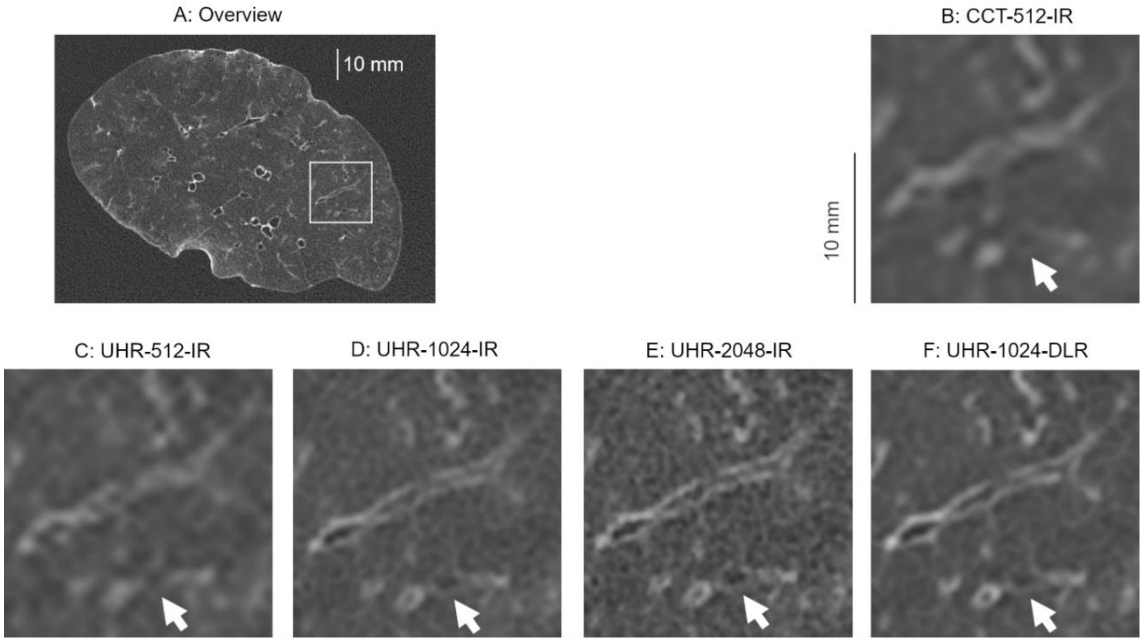

CCT, conventional CT; UHR, ultra-high-resolution CT; IR, iterative reconstruction; DLR, deep learning reconstruction.

The histological image corresponding to the CT images was published in the previous paper (2).

Figure S6: Images showing bronchioles with inner diameters of 519 µm (arrows).

(A) Overview of CT image. (B) Image with an enlarged local area (square in A) on CCT-512-IR, (C) UHR-512-IR, (D) UHR-1024-IR, (E) UHR-2048-IR, and (F) UHR-1024-DLR. The bronchiole (arrows) was obscured on the EID-CT images.

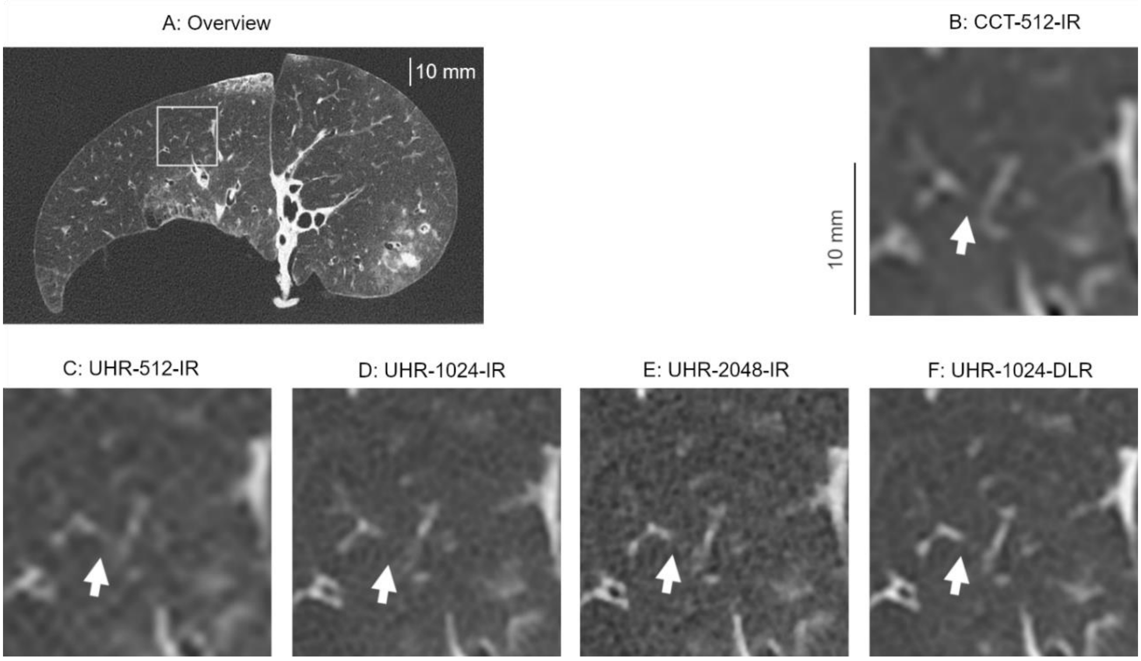

CCT, conventional CT; UHR, ultra-high-resolution CT; IR, iterative reconstruction; DLR, deep learning reconstruction.

The histological image corresponding to the CT images was published in the previous paper (2).

Figure S7: Images showing bronchioles with inner diameters of 1302 μm (arrows).

(A) Overview of CT image. (B) Image with an enlarged local area (square in A) on CCT-512-IR, (C) UHR-512-IR, (D) UHR-1024-IR, (E) UHR-2048-IR, and (F) UHR-1024-DLR. The bronchiole with an inner diameter of 1302 μm (arrows) was obscured on CCT-512-IR, whereas UHR-CT images clearly depict the bronchiole.

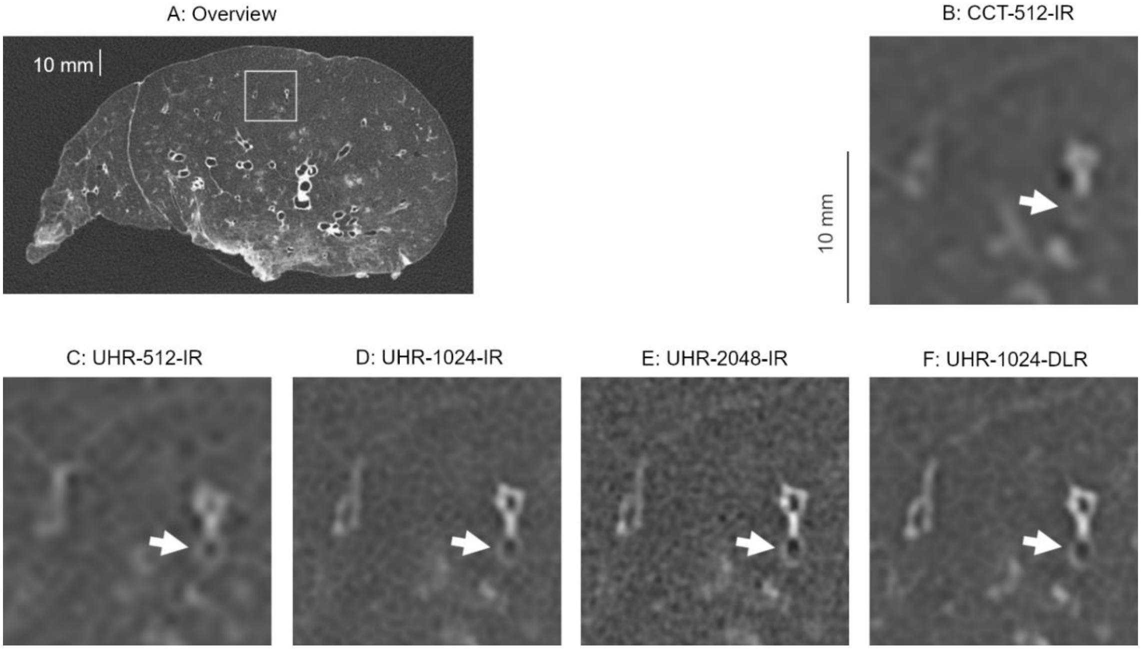

CCT, conventional CT; UHR, ultra-high-resolution CT; IR, iterative reconstruction; DLR, deep learning reconstruction.

The histological image corresponding to the CT images was published in the previous paper (2).

Supplementary Table

Table S1 Reconstruction settings

	CT type	Matrix size	Slice thickness/increment (mm)	IR/DLR
CCT-512-IR	CCT	512	0.5/0.5	IR
UHR-512-IR	UHR-CT	512	0.5/0.5	IR
UHR-1024-IR	UHR-CT	1024	0.25/0.25	IR
UHR-2048-IR	UHR-CT	2048	0.25/0.25	IR
UHR-1024-DLR	UHR-CT	1024	0.25/0.25	DLR
PCD-512-IR	PCD-CT	512	0.6/0.6	IR
PCD-1024-IR	PCD-CT	1024	0.2/0.2	IR

CCT: conventional energy-integrating detector CT; UHR: ultra-high-resolution energy-integrating detector CT; IR: iterative reconstruction; DLR: deep learning reconstruction; PCD: photon-counting detector CT
